# RAB11A-mediated YAP localization to adherens and tight junctions is essential for colonic epithelial integrity

**DOI:** 10.1016/j.jbc.2021.100848

**Published:** 2021-05-29

**Authors:** Sayantani Goswami, Iyshwarya Balasubramanian, Luca D’Agostino, Sheila Bandyopadhyay, Radha Patel, Shail Avasthi, Shiyan Yu, James R. Goldenring, Edward M. Bonder, Nan Gao

**Affiliations:** 1 Department of Biological Sciences, Rutgers University, Newark, New Jersey, USA; 2 Department of Surgery, Epithelial Biology Center, Vanderbilt University School of Medicine, Nashville, Tennessee, USA

**Keywords:** RAB11A, Hippo, YAP, endosomes, epithelial junction, Merlin, colonic regeneration, colitis, DMEM, Dulbecco's modified Eagle's medium, DSS, dextran sulfate sodium, HA, hemagglutinin, HEK293, human embryonic kidney 293, IEC, intestinal epithelial cell, KD, knockdown, LATS1/2, large tumor suppressor 1/2, MST1/2, mammalian sterile 20–like kinase-1/2, YAP, Yes-associated protein

## Abstract

Within the intestinal epithelium, regulation of intracellular protein and vesicular trafficking is of utmost importance for barrier maintenance, immune responses, and tissue polarity. RAB11A is a small GTPase that mediates the anterograde transport of protein cargos to the plasma membrane. Loss of RAB11A-dependent trafficking in mature intestinal epithelial cells results in increased epithelial proliferation and nuclear accumulation of Yes-associated protein (YAP), a key Hippo-signaling transducer that senses cell–cell contacts and regulates tissue growth. However, it is unclear how RAB11A regulates YAP intracellular localizations. In this report, we examined the relationship of RAB11A to epithelial junctional complexes, YAP, and the associated consequences on colonic epithelial tissue repair. We found that RAB11A controls the biochemical associations of YAP with multiple components of adherens and tight junctions, including α-catenin, β-catenin, and Merlin, a tumor suppressor. In the absence of RAB11A and Merlin, we observed enhanced YAP–β-catenin complex formation and nuclear translocation. Upon chemical injury to the intestine, mice deficient in *RAB11A* were found to have reduced epithelial integrity, decreased YAP localization to adherens and tight junctions, and increased nuclear YAP accumulation in the colon epithelium. Thus, RAB11A-regulated trafficking regulates the Hippo–YAP signaling pathway for rapid reparative response after tissue injury.

Coordinated regulation of cell proliferation and differentiation is essential for the maintenance of tissue homeostasis. Cell–cell contact–initiated inhibition of proliferation is a well-characterized mechanism in controlling tissue growth (1). A number of signaling pathways have been linked to contact inhibition of cellular proliferation (2, 3, 4, 5, 6, 7, 8). In tissues such as the intestinal epithelium, such coordinated regulation is of utmost importance because the tissue has a high rate of cell turnover and is constantly bombarded with pathogenic and chemical insults that require rapid repair responses. How distinct, and at times apparently unconnected, pathways are networked and coordinated both within individual cells and across the entire tissue continues to be of intense basic and biomedical research interest (9, 10, 11, 12, 13).

Originally discovered in *Drosophila*, Hippo–Yes-associated protein (YAP) signaling axis was shown to control cell proliferation, growth, and apoptosis (3, 14, 15, 16, 17, 18). The core kinases of the Hippo pathway, mammalian sterile 20–like kinase-1/2 (MST1/2) and large tumor suppressor 1/2 (LATS1/2), along with their respective adaptor proteins, Sav1 and MOB A/B, were found to be sequentially phosphorylated and activated. Activated LATS1/2 phosphorylates YAP at multiple serine/threonine residues (19). Notably, phosphorylation at S127 promotes binding of YAP to 14-3-3, leading to cytoplasmic sequestration. In the absence of S127 phosphorylation, YAP translocates to the nucleus where it acts as a coactivator for various transcription factors, particularly the TEA domain family of transcription factors (16, 20). YAP can also be sequestered in the cytoplasm as part of the β-catenin destruction complex and is released upon Wnt signaling leading to both Wnt and YAP-dependent transcriptional responses (2, 21).

Control of YAP localization is also linked to integrity of cell–cell contacts and actin cytoskeleton contractile activity (22, 23, 24, 25, 26). For example, disruption of adherens junction components E-cadherin and β-catenin results in enhanced nuclear localization of YAP (27), whereas overexpression of E-cadherin leads to decreased nuclear localization and increased cytoplasmic sequestration of YAP1 (2). Also, α-catenin, which is anchored to E-cadherin and β-catenin, was shown to indirectly associate with YAP and modulate the Hippo-signaling pathway (28, 29). Merlin, originally identified as the product of the tumor suppressor gene, *Nf2*, is a component of the apical membrane actin cytoskeleton that has been identified as an upstream regulator of Hippo–YAP signaling pathway (30). In addition to a role in regulating the membrane actin cytoskeleton, Merlin serves as a scaffold for LATS, enabling LATS phosphorylation of YAP (31). Similarly, *motin* family of proteins (angiomotin, AMOT; angiomotin-like protein 1 and 2, AMOTL1, AMOTL2, respectively) function in scaffolding MSTs, LATS, and YAP at tight junction, thereby regulating YAP signaling (32, 33, 34, 35, 36). Thus, it is essential to understand the coordinated regulation of Hippo–YAP signaling in relation to cell–cell contact formation, YAP nuclear localization, and proliferation. This has significance to not only normal tissue maintenance but also tissue repair and pathogenesis (17, 25, 26, 37).

Previously, we reported that genetic ablation of *Rab11a* in mouse or *Drosophila* intestinal epithelial cells (IECs) results in increased nuclear accumulation of YAP, production of inflammatory cytokines, development of epithelial hyperplasia, and elevated tumorigenicity (38, 39). Those reports provided physiological context and extended published work on the protein interactome analysis of the Hippo pathway intersecting with cell–cell junction components and vesicular trafficking regulators (40, 41). Using genetic knockdown (KD) and knockout models in mouse and human colonic epithelial cells, this report examined the consequences of Rab11a activity on coordinated YAP trafficking between cytoplasmic and nuclear compartments and the impact of this positioning on epithelial repair. Interestingly, YAP demonstrates dependencies on Merlin and RAB11A for sequestration and perijunctional localization of YAP. These observations on the spatial assembly and positioning of YAP-associated scaffolds lend insight into trafficking mechanisms regulating YAP signaling and subsequent control of cell proliferation and tissue repair.

## Results

### RAB11A expression determines YAP cellular localization

**Figure 1:**
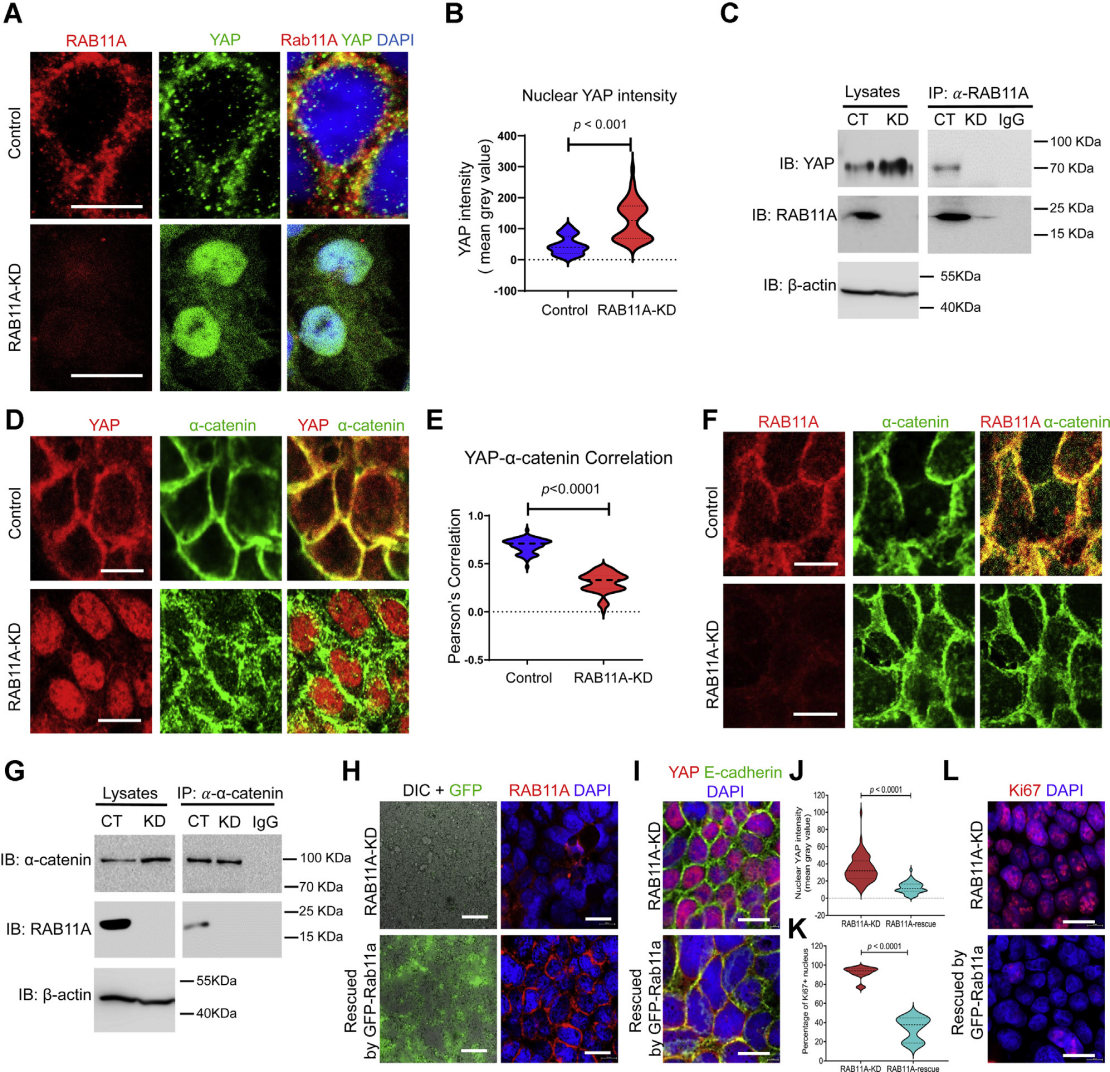
**RAB11A intersects cytoplasmic YAP.***A*, immunofluorescent staining of RAB11A (*red*) and YAP (*green*) in control and *RAB11A*-KD Caco-2 cells. Nuclei were stained by DAPI (*blue*). *B*, quantification of nuclear YAP intensities in control and *RAB11A*-KD Caco-2 cells (n = 50 cells for each genotype). *C*, coimmunoprecipitation (Co-IP) assay for endogenous YAP and RAB11A. Total cell lysates were immunoprecipitated by RAB11A antibody and blotted for YAP. Data represent at least three independent experiments. *D*, immunofluorescent staining of YAP (*red*) and α-catenin (*green*) in control and *RAB11A*-KD Caco-2 cells. *E*, Pearson's correlation between YAP and α-catenin was analyzed along the cell–cell junctions in control cells (*r* = 0.69, n = 65 cells) and *RAB11A*-KD cells (*r* = 0.3, n = 65 cells). *F*, immunofluorescent staining for RAB11A (*red*) and α-catenin (*green*). *G*, co-IP assay between endogenous α-catenin and RAB11A in control and *RAB11A*-KD cells. *H*, a *RAB11A*-KD rescue cell line expressed a GFP-tagged mouse Rab11a resistant to shRNA silencing. The bright field and GFP were imaged from live cells. The rescued cells were fixed and stained positive for RAB11A (*red*). *I*, immunofluorescent staining for YAP (*red*) and E-cadherin (*green*) in fixed *RAB11A*-KD and rescued cell lines. *J*, quantification of nuclear YAP intensities in *RAB11A*-KD (n = 78 cells) and rescued Caco-2 monolayers (n = 52 cells). *K*, quantification of percentage of Ki67-postive cells in fixed *RAB11A*-KD (eight different fields) and rescued Caco-2 monolayers (six different fields). *L*, representative images for *K*. The scale bars represent 10 μm. DAPI, 4′,6-diamidino-2-phenylindole; KD, knockdown; YAP, Yes-associated protein.

Previously, we reported that in IECs, RAB11A expression effected both the expression levels of YAP and its nuclear localization (39). Monolayer cultures of both control and *RAB11A*-KD Caco-2 cells were stained by indirect immunofluorescence for YAP and RAB11A, imaged by laser scanning confocal microscopy. YAP was observed on numerous fluorescent puncta with an observed localization to the periphery of intercellular junctional region (Fig. 1*A*). RAB11A staining identified a similar cytoplasmic localization of small fluorescent cytoplasmic puncta. Pearson's correlation analysis identified that YAP and RAB11A were colocalized in some subpopulation of these puncta (Fig. 1*A*, correlation quantified in Fig. S1*A*). *RAB11A*-KD resulted in reduction of cytoplasmic and peripheral YAP while the fluorescent staining for YAP was dramatically increased in nuclei (Fig. 1*A*, nuclear YAP quantified in Fig. 1*B*). Immunoprecipitation assays of cell lysates from control and *RAB11A*-KD Caco-2 cell lines using anti-RAB11A antibodies resulted in coprecipitation of YAP with endogenous RAB11A (Fig. 1*C*). More specifically, RAB11A coprecipitated with YAP that was phosphorylated on S127 (Fig. S1*C*), a key cytoplasmic retention signal. The phosphorylated YAP (S127) was diminished upon depletion of RAB11A, consistent with our previous report (39).

The observed colocalization of RAB11A and YAP opened the possibility that RAB11A may contribute to Hippo–YAP signaling and its reliance on cell–cell contacts for tension sensing (2, 25, 27, 32, 33, 42). A reasonable supposition given the adherens junction protein α-catenin was shown to bind YAP and regulates YAP phosphorylation status and cytoplasmic retention (29). In control Caco-2 cell monolayers, α-catenin possessed distinct and sharp cell–cell junction staining with a lesser degree of diffuse cytoplasmic perijunctional staining (Fig. 1*D*, correlation with RAB11A quantified in Fig. S1*B*). *RAB11A*-KD in Caco-2 cells resulted in a diffused labeling of cell–cell contacts for α-catenin, which was now observed as scattered fluorescence staining throughout the cytoplasm (Fig. 1, *D* and *F*). Similar results were obtained in preparations of immunostained *Rab11a*-deficient enteroids and mouse intestinal epithelia (Fig. S1*D*). In control Caco-2 cells, Pearson's correlation analysis comparing localization of YAP and α-catenin identified a significant correlation (*r* = 0.69) in control cells compared with a diminished correlation (*r* = 0.3) in *RAB11A*-KD cells (Fig. 1, *D* and *E*). By indirect immunofluorescence, RAB11A and α-catenin were colocalized to cell junctions in Caco-2 cells (Fig. 1*F*) as well as in mouse enteroids (Fig. S1*E*). Protein levels of α-catenin in *RAB11A*-KD Caco-2 cells were slightly elevated, when compared with control cells, and RAB11A was coimmunoprecipitated by α-catenin antibody pulldowns (Fig. 1*G*). We further analyzed YAP localization in a rescued *RAB11A*-KD Caco-2 cell line, where a mouse *Rab11a* that was resistant to the human *RAB11A*-targeting shRNA was expressed (Fig. 1*H*). Re-expression of mouse Rab11a redistributed the nuclear YAP to intercellular junctions (Fig. 1, *I* and *J*). In addition, the high index of proliferation marked by Ki67 in *RAB11A*-KD cells, as we previously reported (39), was also significantly reduced in the rescued cell line (Fig. 1, *K* and *L*). Thus, RAB11A has a pronounced impact on the localization of YAP and the adherens junction component, α-catenin.

### RAB11A regulates YAP association with adherens junction components

**Figure 2:**
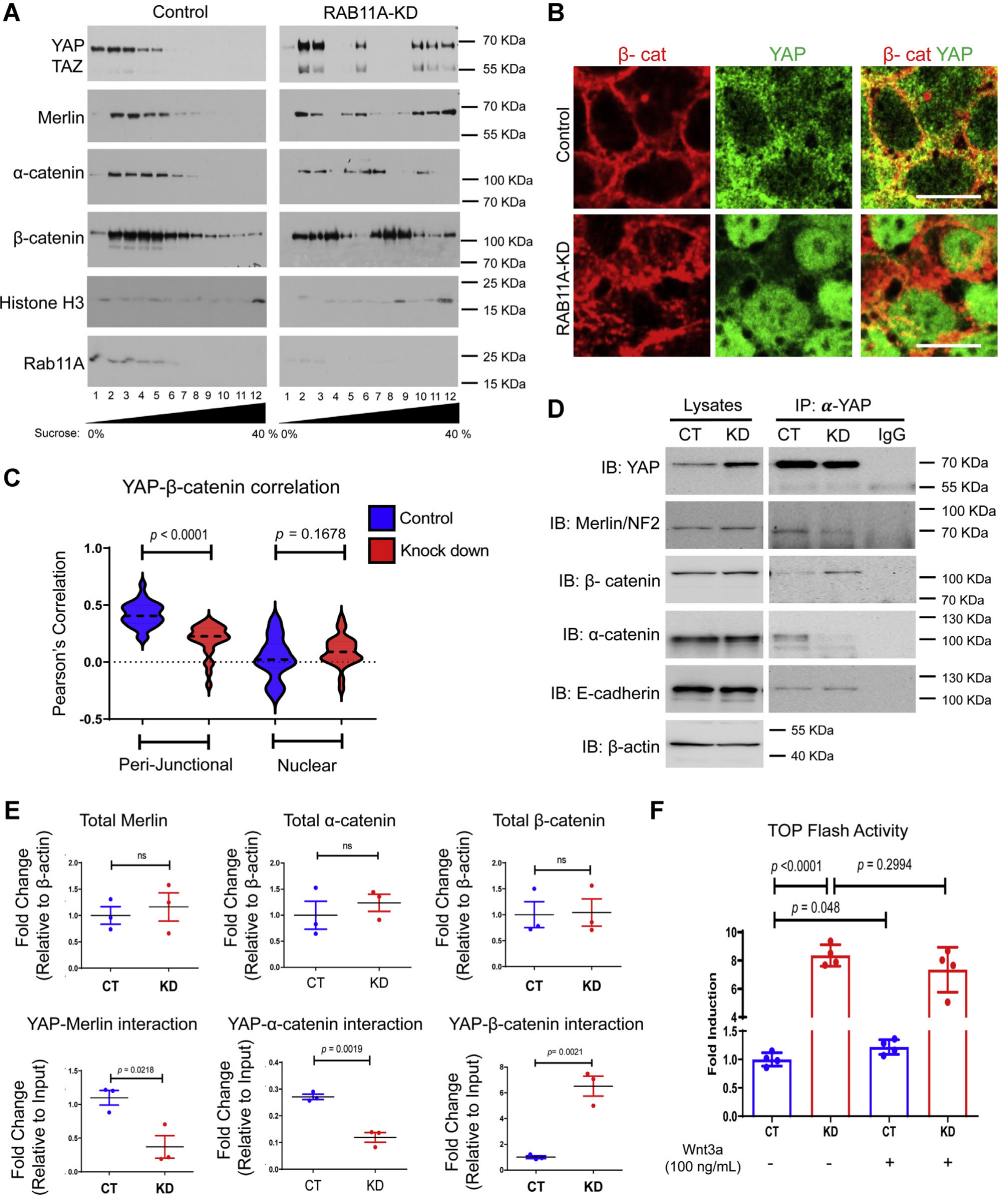
**Impact of RAB11A deficiency on YAP association with junction.***A*, subcellular compartmentalization of YAP was determined by sucrose gradient sedimentation assay using control and *RAB11A*-KD total lysates. Subcellular fractions (lane 1–12: 0–40% sucrose fractions) were resolved and blotted by antibodies against YAP/TAZ, Merlin, α-catenin, and β-catenin. Histone H3 was used to identify the nuclear fraction. *B*, immunofluorescent staining of YAP (*green*) and β-catenin (*red*) in control and *RAB11A*-KD cells. Note the presence of colocalization in nucleus of *RAB11A*-KD cells. *C*, Pearson's correlation analysis of YAP–β-catenin at junctional and in nuclear compartment was conducted in control and *RAB11A*-KD cells (n = 28 for each genotype). *D*, coimmunoprecipitation assay between YAP and adherens junctional proteins (Merlin, β-catenin, α-catenin, and E-cadherin) in control and *RAB11A*-KD cells. *E*, quantification of total Merlin, β-catenin, α-catenin, as well as their interaction with YAP. Each assay was performed in three independent experiments. *F*, TOP-Flash reporter assay was performed in control and *RAB11A*-KD cells that were unstimulated or stimulated by Wnt3a (100 ng/ml). Data represent four independent experiments. The scale bars represent 10 μm. TAZ, transcriptional coactivator with PDZ-binding motif; YAP, Yes-associated protein.

To dissect the underlying mechanism of YAP localization, control and *RAB11A*-KD Caco-2 cells were subjected to cellular fractionation, density gradient centrifugation, and immunoblotting to assess intracellular compartmentalization of YAP and adherens junction components. In control cells, YAP and RAB11A cosedimented in low-density fractions (nos. 1–5; Fig. 2*A*). These fractions were also enriched for α-catenin and β-catenin (Fig. 2*A*) as well as GRP78 (endoplasmic reticulum), Gm130 (Golgi), and Rab7 (lysosome) (Fig. S2*A*). The actin membrane cytoskeleton scaffolding protein, Merlin, was also detected in these fractions (Fig. 2*A*). YAP, Merlin, α-catenin, and RAB11A were repeatedly observed in the top half of the sucrose gradients and diminished in the nucleus bottom fraction enriched for the nuclear marker, histone H3. A small amount of β-catenin was observed in the densest fractions correlating with nuclear compartment.

In *RAB11A*-KD cells, the sedimentation profiles of YAP changed, and it was now observed in three distinct regions of the gradient. The top lowest density quarter of the gradient contained YAP, and the same fractions contained a trace amount of residual RAB11A (Fig. 2*A*). YAP was also detected in the densest nuclear-containing fractions as identified by histone H3 (Fig. 2*A*), an observation consistent with the nuclear YAP immunofluorescent localization (Figs. 1, *A*, *B*, and *D* and 2*B*). There was also a similar sedimentation towards denser gradients for both β-catenin and Merlin (Fig. 2*A*). Immunofluorescence imaging identified YAP–β-catenin perijunctional colocalization in control Caco-2 cells (Fig. 2, *B* and *C*). In *RAB11A*-KD cells, a loss of such perijunctional colocalization corresponded to a detectable increase of YAP–β-catenin nuclear localization (Fig. 2, *B* and *C*).

The changes in colocalization of YAP with adherens junction proteins were further demonstrated by coimmunoprecipitation analysis using anti-YAP antibodies. Merlin, α-catenin, β-catenin, and E-cadherin were all immunoprecipitated with YAP in control and *RAB11A*-KD Caco-2 cells (Fig. 2*D*, quantified in Fig. 2*E*). Interestingly, upon *RAB11A*-KD, YAP association with Merlin or α-catenin showed significant reduction (Fig. 2, *D* and *E*); YAP association with β-catenin significantly increased (Fig. 2, *D* and *E*), whereas YAP association with E-cadherin remained unchanged (Fig. 2, *D* and *E* and Fig. S2*B*). These changed interactions were not because of elevated total YAP, as precipitated YAP abundance was equivalent in control and *RAB11A*-KD samples (Fig. 2*D* and Fig. S2*B*).

*RAB11A*-KD Caco-2 cells had 22% increase in TEA domain report activities (39). Increased YAP–β-catenin association and nuclear localization correlated with a modified physiological response that was detected by an eightfold higher basal activity of TOP-Flash Wnt reporter in *RAB11A*-KD cells under serum-starved condition, that is, the absence of Wnt ligand (Fig. 2*F*) (43). While Wnt3a ligand treatment of control Caco-2 cells elicited an increase in TOP-Flash activity, there was no detectable effect of Wnt treatment on *RAB11A*-KD cells (Fig. 2*F*). Taken together, these data suggest that RAB11A modulates the adherens junctions, which in turn dictate YAP cytoplasmic retention or nuclear localization.

### RAB11A is required for YAP recruitment to the adherens junction

**Figure 3:**
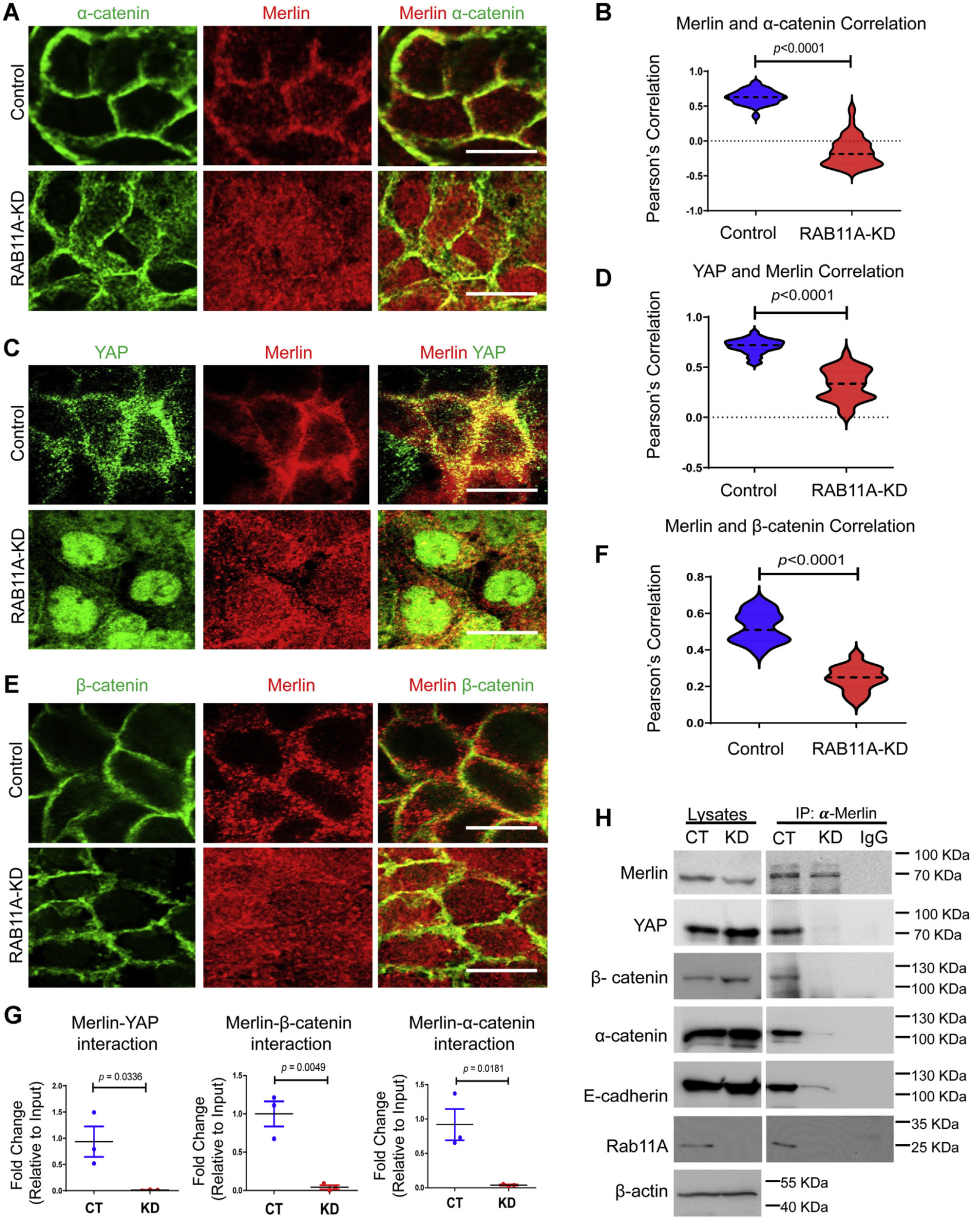
**RAB11A is essential for Merlin localization and interaction with YAP.***A*, immunofluorescent staining of Merlin (*red*) and α-catenin (*green*) in control and *RAB11A*-KD cells. *B*, Pearson's correlation between Merlin and α-catenin was conducted along the cell–cell junction in control cells (*r* = 0.62, n = 54 cells) and *RAB11A*-KD cells (*r* = −0.15, n = 54 cells). *C*, immunofluorescent staining of Merlin (*red*) and YAP (*green*) in control and *RAB11A*-KD cells. *D*, Pearson's correlation between Merlin and YAP was conducted along the cell–cell junction in control cells (*r* = 0.71, n = 30 cells) and *RAB11A*-KD cells (*r* = 0.33, n = 30 cells). *E*, immunofluorescent staining of Merlin (*red*) and β-catenin (*green*). *F*, Pearson's correlation between Merlin and β-catenin was conducted along the cell–cell junction in control cells (*r* = 0.52, n = 15 cells) and *RAB11A*-KD cells (*r* = 0.24, n = 15 cells). *G* and *H*, coimmunoprecipitation assay between Merlin and YAP, α-catenin, β-catenin, and E-cadherin was performed in control and *RAB11A*-KD cells and quantified from three independent experiments. The scale bars represent 10 μm. KD, knockdown; YAP, Yes-associated protein.

The observation of a RAB11A-dependent association between YAP and Merlin may provide mechanistic insight into membrane trafficking and its role in junctional scaffolding and Hippo–YAP signaling (44). We next examined the impact of RAB11A on Merlin association with adherens junction components and the resultant impact on Hippo–YAP effector proteins. In control cells, Merlin was perijunctionally localized with α-catenin (Fig. 3, *A* and *B*) and YAP (Fig. 3, *C* and *D*); this was in agreement with the localization of α-catenin–YAP (Fig. 1, *D* and *E*). In *RAB11A*-KD cells, Merlin was dramatically dispersed throughout the cytoplasm (Fig. 3, *A*, *C*, and *E*). Fluorescence colocalization between Merlin and α-catenin or YAP or β-catenin was significant in control Caco-2 cells compared with their colocalization in *RAB11A*-KD Caco-2 cells (Fig. 3, *B*, *D*, and *F*). Disrupted Merlin localization was also observed in *Rab11a*-deficient mouse enteroids (Fig. S3*A*).

In control Caco-2 cells, anti-Merlin antibodies precipitated Merlin along with YAP, β-catenin, α-catenin, and E-cadherin (Fig. 3*H*, quantified in Fig. 3*G* and Fig. S3*B* for E-cadherin), consistent with the immunofluorescence by anti-YAP (Fig. 2, *D* and *E*). In *RAB11A*-KD cells, the amounts of YAP, β-catenin, and α-catenin coprecipitated were significantly reduced than that for controls (Fig. 3*G*). We noted that the trend of relative amounts of individual proteins precipitated was consistent with the results obtained using anti-YAP antibodies (please compare with Fig. 2*D*). To determine the requirement of active RAB11A in forming the RAB11A–Merlin complex, cells were transfected with a dominant-negative Flag-RAB11A (S25N), or Flag-wildtype RAB11A, along with hemagglutinin (HA)-tagged Merlin. Pulldown using anti-Flag antibodies detected a reduction in the amount of HA-tagged Merlin coprecipitating with dominant-negative RAB11A as compared with wildtype RAB11A (Fig. S3, *C* and *D*).

### Merlin depletion recapitulated impact of RAB11A deficiency

**Figure 4:**
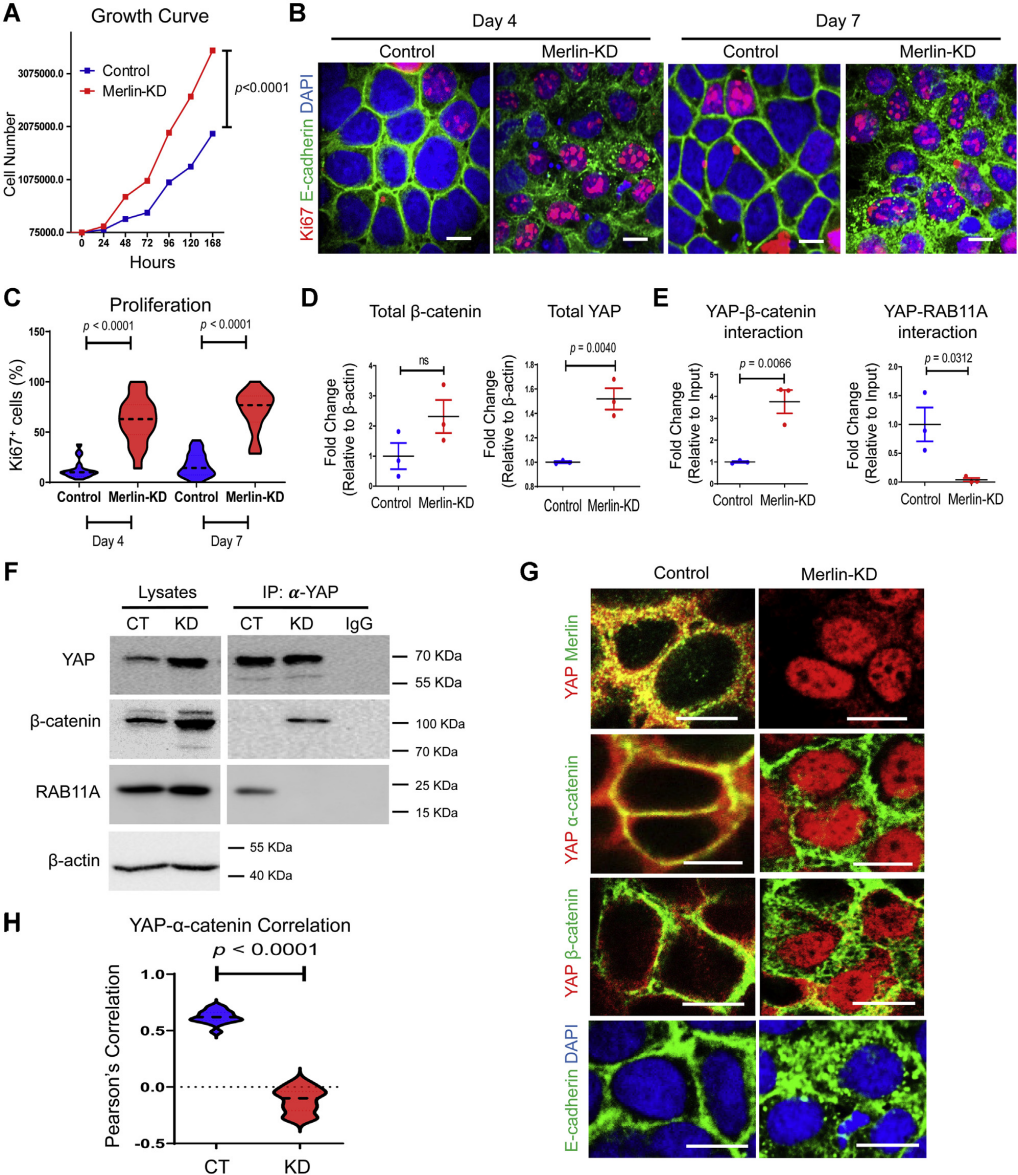
**Merlin depletion disrupts junctional YAP localization and recapitulates RAB11A-KD defects.***A*, cell growth curves of control and *Merlin*-KD Caco-2 cells, representing three independent experiments. *B*, immunostaining for Ki67 was performed in control and *Merlin*-KD cells at indicated time points. Nuclei were stained by DAPI. *C*, quantification of Ki67+ nucleus (%) on day 4 and 7 was performed for control and *Merlin*-KD cells (20 independent fields for each genotype). *D*–*F*, co-IP assays between YAP and β-catenin or RAB11A were performed in control and *Merlin*-KD cells and quantified from three independent experiments. *G*, immunofluorescent staining of YAP and Merlin, α-catenin, β-catenin, and E-cadherin in control and *Merlin*-KD cells. *H*, Pearson's correlation between YAP and α-catenin was analyzed along cell–cell junction in control cells (r = 0.62, n = 15 cells) and *Merlin*-KD cells (r = −0.11, n = 15 cells). The scale bars represent 10 μm. Data represent at least three independent experiments. DAPI, 4′,6-diamidino-2-phenylindole; KD, knockdown; YAP, Yes-associated protein.

The aforementioned results suggested that Merlin associates with physiologically functional RAB11A and that RAB11A may regulate the Hippo–YAP pathway involving Merlin. To further address potential RAB11A–Merlin functionality, shRNA was used to establish stable Caco-2 cell lines deficient in Merlin (Fig. S4, *A* and *B*). *Merlin*-KD cells exhibited increased cellular proliferation as determined by cell growth rate (Fig. 4*A*) or numbers of Ki67+ cells (Fig. 4, *B* and *C* and Fig. S4*C*). Compared with control cells forming monolayer, *Merlin*-KD cells tend to grow on top of each other (Fig. S4*D*), suggesting that the cells might no longer be contact inhibited.

We thus examined adherens junction components in *Merlin-KD* cells. Merlin-depleted cells contained elevated total YAP and β-catenin (Fig. 4, *D* and *F*). Lysates from *Merlin*-KD Caco-2 cells were subjected to immunoprecipitation using anti-YAP antibodies. *Merlin* KD resulted in increased YAP associations with β-catenin (Fig. 4, *E* and *F*). Interestingly, in the absence of Merlin, RAB11A was no longer immunoprecipitated with YAP (Fig. 4, *E* and *F*). These changes were not because of altered total RAB11A (Fig. 4*F*, quantified in Fig. S4*E*) or the abundance of YAP being precipitated (Fig. 4*F* and Fig. S4*F*).

By indirect immunofluorescence localization in *Merlin*-KD Caco-2 cells, YAP was predominantly localized inside the nucleus (Fig. 4*G*). While there were notable perturbations in α-catenin, β-catenin, and E-cadherin junctional positioning (Fig. 4*G* and Fig. S5, *A*–*C*), YAP correlation with α-catenin was diminished on the basis of Pearson's analysis (Fig. 4*H*). In *Merlin*-KD cells, E-cadherin appeared to form large puncta dispersed throughout the cytoplasm possibly representing stalling of trafficking within a membraneous compartment (Fig. 4*G*).

### RAB11A deficiency affects YAP–AMOTL-2 association

**Figure 5:**
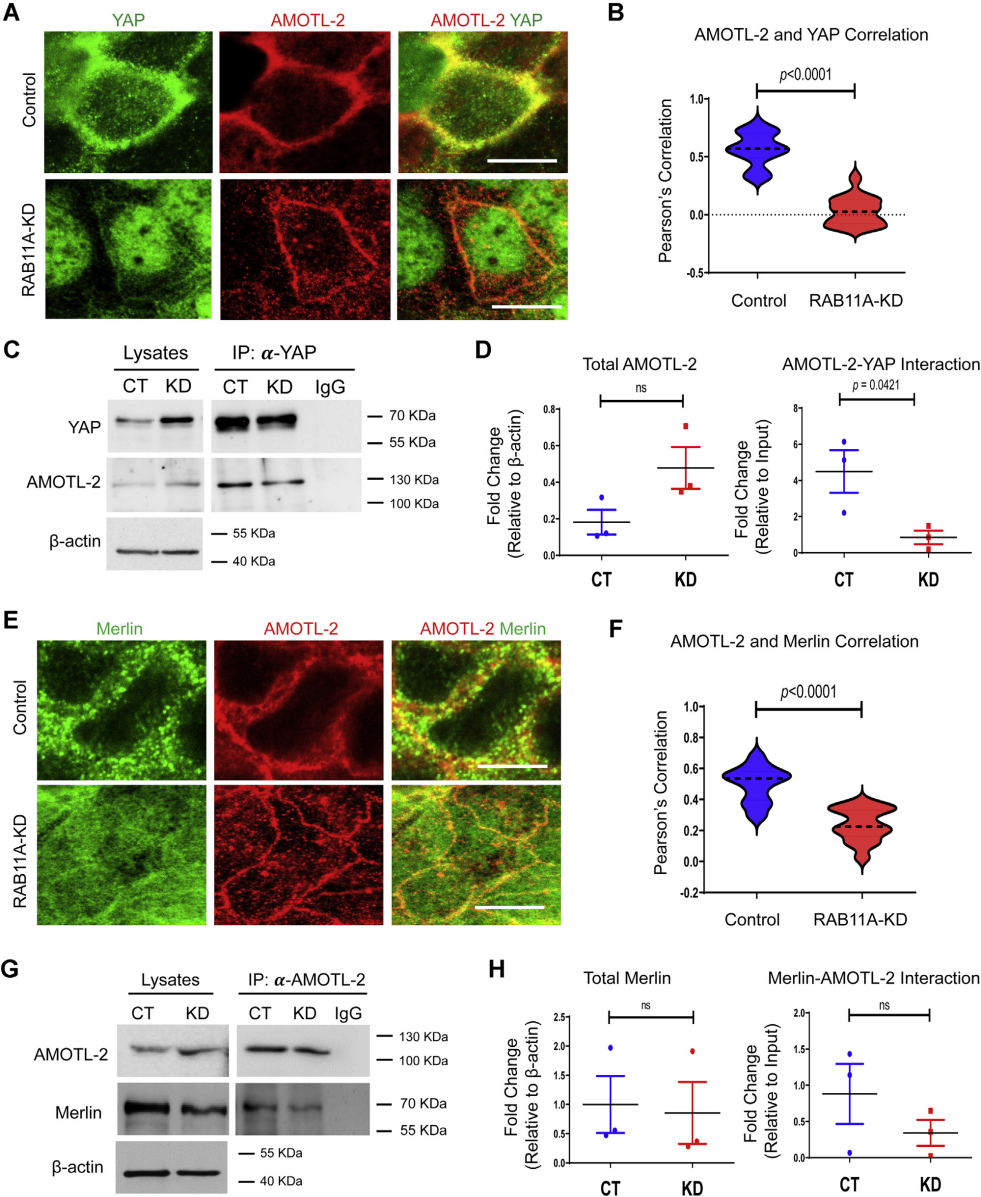
**RAB11A deficiency alters AMOTL-2 and YAP association.***A*, immunofluorescent staining of YAP (*green*) and AMOTL-2 (*red*) in control and *RAB11A*-KD cells. In *RAB11A*-deficient Caco-2 cells, YAP was mostly nuclear with AMOTL-2 retaining its junctional localization. *B*, Pearson's correlation between AMOTL-2 and YAP along the cell–cell junction in control cells (*r* = 0.57, n = 30 cells) and *RAB11A*-KD cells (*r* = 0.03, n = 30 cells). *C* and *D*, coimmunoprecipitation assay between AMOTL-2 and YAP was performed in control and *RAB11A*-KD Caco-2 cells and quantified from three independent experiments. *E*, immunofluorescent staining of Merlin (*green*) and AMOTL-2 (*red*) was performed in control and *RAB11A*-KD cells. *F*, Pearson's correlation between AMOTL-2 and Merlin was analyzed along the cell–cell junction in control cells (*r* = 0.49, n = 30 cells) and *RAB11A*-KD cells (*r* = 0.23, n = 30 cells). *G* and *H*, coimmunprecipitation assay between AMOTL-2 and Merlin was performed in control and *RAB11A*-KD cells and quantified from three independent experiments. The scale bars represent 10 μm. KD, knockdown; YAP, Yes-associated protein.

The aforementioned results indicated that YAP is associated with a potential RAB11A–Merlin–adherens component complex that jointly modulates cytoplasmic and nuclear localization of YAP. In particular, phosphorylation-independent sequestration of YAP *via* Merlin depends upon the presence of the junctional scaffolding angiomotin/*motin* (Amot, Amotl1, and Amotl2) family of proteins (33, 45). Consistent with previous report that AMOTL-2 colocalizes with RAB11 (46), and complexes with YAP (33), we found that YAP and AMOTL-2 were colocalized to the junctional periphery of cell–cell contacts in control Caco-2 cells, and the colocalization was reduced in *RAB11A*-KD Caco-2 cells (Fig. 5, *A* and *B*). In *RAB11A*-KD cells, a portion of AMOTL-2 staining retained sharp junctional localization and diffuse punctate staining in the cytoplasm (Fig. 5*A*). AMOTL-2 was coprecipitated using anti-YAP antibodies, and the degree of coprecipitation was reduced in *RAB11A*-KD cells (Fig. 5, *C* and *D*). Similar results were obtained when we examined Merlin and AMOTL-2 localization and association (Fig. 5, *E* and *F*). This RAB11A-dependent association was also suggested by immunoprecipitation and immunoblotting (Fig. 5, *G* and *H* and Fig. S5*D*), but the overall impact of RAB11A deficiency on AMOTL-2 was not as great as the impact on the association of YAP with Merlin or junctional proteins. These data in aggregate support a role played by RAB11A membrane sorting unit in regulating YAP complexing with a scaffold of junction-associated proteins to control its cytoplasmic retention.

### Rab11a-regulated YAP localization is critical for mouse colonic epithelial repair

**Figure 6:**
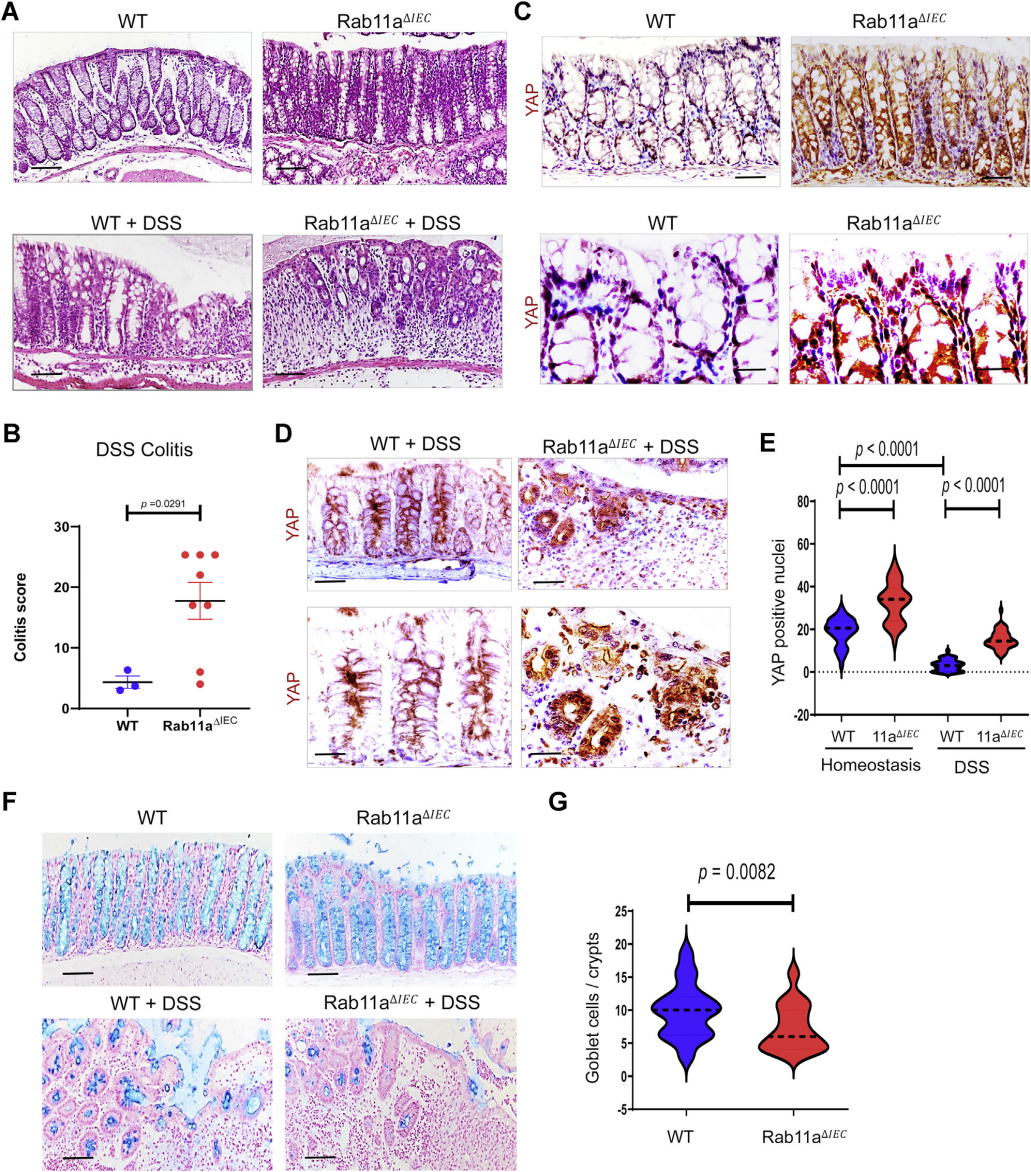
**Rab11a regulates YAP localization and colonic epithelial repair during experimental colitis.***A*, histology analysis of wildtype and *Rab11a*^*ΔIEC*^ mice in homeostasis and after 3% DSS treatment. DSS-treated *Rab11a*^*ΔIEC*^ mice exhibited a pronounced epithelial damage compared with their wildtype counterparts. *B*, colitis scoring of DSS-treated wildtype and *Rab11a*^*ΔIEC*^ mice based on three different litters. Note that 25% of *Rab11a*^*ΔIEC*^ mice died during the DSS treatment. *C*, immunohistochemistry for YAP was performed in wildtype and *Rab11a*^*ΔIEC*^ colonic epithelium in homeostasis. *D*, YAP staining was performed on DSS-treated mouse colonic epithelium. Note that DSS-treated wildtype colonic epithelium showed junctional YAP, whereas *Rab11a*^*ΔIEC*^ colonic epithelium exhibited a significant increase in YAP-positive nuclei. *E*, numbers of YAP-positive nuclei were quantified from wildtype and *Rab11a*^*ΔIE*C^ colonic glands in homeostasis and after DSS treatment. Data were quantified from 20 crypts per section for each mouse. *F*, colonic sections of wildtype and *Rab11a*^*ΔIEC*^ mice, in homeostasis and after DSS treatment, were stained with Alcian blue. *G*, DSS-treated *Rab11a*^*ΔIEC*^ mice exhibited a significant decrease in the number of Alcian blue–positive goblet cells, compared with its wildtype counterpart. Data were quantified from 15 crypts per section for each mouse. The scale bars represent 10 μm. DSS, dextran sulfate sodium; YAP, Yes-associated protein.

YAP is required for colonic epithelial repair (47, 48). To address the physiological relevance of Rab11a-dependent trafficking of YAP, wildtype and *Rab11a*^ΔIEC^ mice were challenged with 3% dextran sulfate sodium (DSS) to induce colonic epithelial junction injury (49). Compared with wildtype mice, DSS-treated *Rab11a*^ΔIEC^ mice exhibited distorted colonic morphology with notable loss of regular spacing and positioning of crypts along the epithelium (Fig. 6*A*) and were identified to have a significant increase in colitis pathology score (Fig. 6*B*) compared with the wildtype littermates.

In wildtype mice, YAP was detected at junction and in the nuclei of colonic crypt cells in homeostasis (Fig. 6*C*), consistent with previous reports (47). Upon DSS treatment, there was a decrease in nuclear staining intensity, and a prominent cytoplasmic and junctional staining for YAP (Fig. 6*D*, quantified in Fig. 6*E*). In *Rab11a*^*ΔIEC*^ mice, intensity of nuclear staining of YAP and number of YAP-positive crypt nuclei increased in homeostasis (Fig. 6*C*). DSS-treated *Rab11a*^*ΔIEC*^ mice showed elevated YAP levels and nuclear localization (Fig. 6*D* quantified in Fig. 6*E*).

Proper epithelial differentiation is a hallmark of normal colonic tissue repair. We found a significant decrease in the number of goblet cells, as determined by Alcian blue staining, in DSS-treated *Rab11a*^*ΔIEC*^ mice when compared with its wildtype counterpart (Fig. 6*F*, quantified in Fig. 6*G*). This defective goblet cell differentiation was not a consequence of Rab11a deficiency (please see homeostasis condition, Fig. 6*F*). Thus, the presence of Rab11a traffic in colonic epithelia promoted tissue repair and epithelial differentiation, by facilitating junctional YAP localization, and in so doing may have favored epithelial differentiation over continued proliferation.

## Discussion

Hippo signaling is a cellular monitoring system that enables cells and tissues to appropriately respond to multiple stimulatory inputs ranging from growth factors, physical tension along cell–cell and cell–substrate contacts, and injury (50, 51, 52, 53, 54, 55). Dysregulation in sensing or responding to one or multiple of the inputs can result in uncontrolled cell growth often leading to abnormal tissue development and potentially disease progression (56, 57). YAP signaling is a central component of the sensing pathway, and its activity is predominantly determined by post-translational modification resulting in cytoplasmic retention or translocation into the nucleus, where it functions as a transcriptional cofactor. Cytoplasmic retention or nuclear localization of YAP is somewhat akin to the Wnt signaling pathway (21).

RAB11 family members are essential regulatory constituents of the recycling endosome. Rab11 and its interacting proteins have been extensively studied in maintenance of cellular trafficking, cell polarity, and junctional integrity; for example, the interaction of RAB11 with RAB11-family-interacting protein 2 is required for Myo-5 trafficking, and phosphorylation of RAB11-family-interacting protein 2 participates in establishing apical cell polarity (38, 58, 59, 60, 61, 62, 63). Previously, we demonstrated that loss of Rab11a in the human, mouse, and fly gut epithelium leads to hyperproliferation, increased tumorigenic activity, and progression of colon cancer (38, 39). These changes in intestinal epithelial homeostasis and health were coincident with an increase in intracellular YAP abundance and nuclear localization (39). We hypothesized that RAB11A endosomes are required for monitoring tumorigenic signaling and can thus impact colonic tumorigenesis and cancer development. In the current report, we dissected the possible molecular mechanism by which RAB11A controls the YAP signaling pathway.

**Figure 7:**
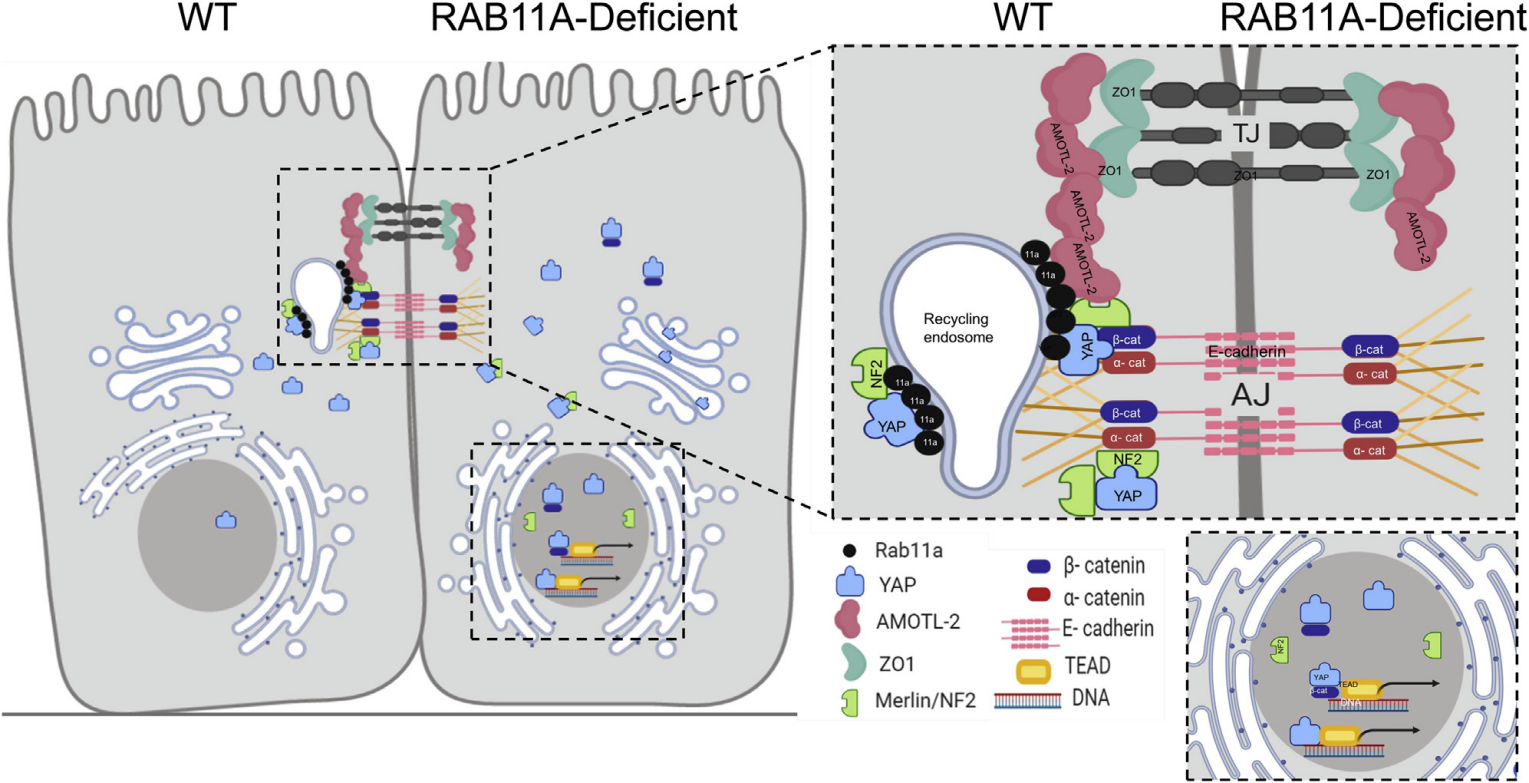
**A schematic model of RAB11A delivery of YAP to epithelial junction.** RAB11A endosomes may provide a pedestal for the assembly of the key YAP regulatory components, regulating the localization and signaling activity of YAP. At homeostasis, RAB11A vesicles associate with YAP, its junctional binding partners: α-catenin, Merlin/NF2, β-catenin, and AMOTL-2, consecutively forming a signaling endosome. YAP in this complex potentially subjects to LATS regulation, by the virtue of its proximity to junctionally localized LATS kinases, leading to sequestration in the cytoplasm. The negative regulators of YAP, including Merlin, are capable of sequestering YAP at cell–cell junctional complexes. In the absence of RAB11A, this platform for signaling phosphorylation and sequestration is lost. Unphosphorylated YAP, either by itself or bound to β-catenin, enters the nucleus triggering a plethora of downstream events promoting cell proliferation. LATS, large tumor suppressor; YAP, Yes-associated protein. (This image has been created using BioRender.com).

Important to the YAP regulatory networks are interactions with various components of cell–matrix and cell–cell adhesion complexes (24, 50, 64). How these various interactions are integrated and spatially coordinated remains of intense study. In this report, we detailed the existence of protein complexes composed of RAB11A, YAP, adherens junction proteins (α-catenin), Merlin, and AMOTL-2, essential Hippo signaling partners, and actin membrane cytoskeletal scaffolding proteins. Loss of RAB11A led to reduced phosphorylation of YAP at S127 (39), a post-translational modification that drives cytoplasmic sequestration. This observation indicates that RAB11A function intersected with Hippo signaling by affecting LATS kinase activity that in turn controls nuclear YAP. Loss of RAB11A was also associated with altered localization of adherens junction proteins and their association with YAP. Coordinated temporal localization of YAP–β-catenin to nuclei is likely to, in part, account for the documented increased proliferation in RAB11A-deficient cells. Thus, apical RAB11A vesicular trafficking may guide formation of a junctionally positioned Hippo–YAP signalosome capable of rapid response to changes in cell–cell contact, tissue tension, and extracellular cues (Fig. 7).

The principle of this model was tested *in vivo* using the experimental colitis that impairs junctional integrity of the intestinal epithelium (65). Epithelial damage triggers repair mechanisms, which entail a fine balance between cellular proliferation and differentiation within a tissue that under conditions of normal homeostasis is in a constant state of cell and tissue renewal (66, 67, 68, 69, 70, 71). Cell–cell junctional proteins, such as α-catenin, β-catenin, Merlin, and AMOTL-2, form a junctional complex that regulates YAP sequestration at the junctions with nuclear YAP needed for proliferation and tissue renewal. DSS treatment results in disruption of junctional and tissue integrity that induces cellular proliferation followed by proper differentiation as the normal epithelium mounts the repair pathway to restore junctional and barrier integrity. By comparison, because of an enhanced YAP nuclear localization and failed junctional delivery of YAP upon tissue damage, DSS-treated Rab11a-deficient mice had decreased epithelial differentiation and impaired epithelial restoration, leading to exacerbated colitis. We propose that upon epithelial barrier injury in a wildtype tissue context, loss of adherens junction integrity triggers YAP nuclear translocation and cell proliferation, which are attenuated by RAB11A-mediated delivery of YAP to junction for epithelial differentiation and re-establishment of the damaged junctions after the tissue reaches sufficient cell number. Thus, as the epithelial contacts, or apical junctions, mature, the tissue undergoes contact inhibition by RAB11-dependent recompartmentalizing YAP to the cytoplasm and junction under the guidance of Merlin. In the absence of RAB11A or Merlin, there is a diminished capacity to retain YAP to junction resulting in an improper tissue repair.

The RAB11A endosome may serve as a point of convergence between different signaling pathways. For example, YAP as a component of the β-catenin destruction complex is also capable of regulating cellular proliferation through the Wnt–β-catenin signaling pathway (21). Thus, we described mechanisms by which RAB11A directs YAP trafficking for cytoplasmic retention. Further, these studies provide a link among apical junctional components, Hippo-YAP, and Wnt–β-catenin signaling. In principle, RAB11A endosomes would serve as a membranous compartment to regulate signaling processes for mature epithelial homeostasis (72, 73, 74).

## Experimental procedures

### Mice

*Rab11a*^*flox/flox*^, *Villin-Cre*, and *Villin-CreER* mice have been described previously (38, 75, 76). All animal experiments were performed on littermates and carried out in an Association for Assessment and Accreditation of Laboratory Animal Care International–accredited animal facility at Rutgers University, Newark. The procedures were approved by the Rutgers University Institutional Animal Care and Use Committee.

### DSS-induced experimental colitis

Wildtype and Rab11a^ΔIEC^ mice were administered with 3% DSS (colitis grade, 36–50 KDa, SKU 0216011080; MP Biologics) in tap water for 8 days. Colon tissue samples were harvested, and colonic damage was scored blindly as described previously (65). Scores (0–4) were assigned based on the severity of epithelial injury and leukocyte infiltration into the mucosa, submucosa, and muscularis. These three scores were multiplied by an extended factor to assess the extent of the change: one for focal, two for patchy, and three for diffuse, and summed to achieve the final score out of the maximal score of 36. At least three independent litters were used for the experiments.

### Culture of human Caco-2 cell lines

Human colon adenocarcinoma cell line, Caco-2, was purchased from American Type Culture Collection and cultured in Dulbecco's modified Eagle's medium (DMEM) 1×, supplemented with 20% fetal bovine serum and 1% penicillin–streptomycin. Specific shRNA designed against RAB11A and Merlin was delivered by lentivirus particles, as described (77). The efficiency of the generated KD Caco-2 cells was confirmed by Western blotting for RAB11A and Merlin (38, 39). KD and control cell lines were maintained in medium containing puromycin ranging from 2 to 12.5 μg/ml. The rescue of Caco-2 *RAB11A*-*KD* by a mouse *Rab11a* resistant to the *RAB11A*-silencing shRNA was described by us previously (78).

### Mouse intestinal enteroid culture and embedding

Crypt isolation and enteroid culture was carried out as per the procedure described (38, 39), originally adapted from a report by Sato and Clevers (79). Wildtype and Rab11a^flox/flox^;Villin-CreER mice of 10 to 12 weeks of age were sacrificed, and their small intestines were collected. The intestinal samples were further cut into smaller pieces and washed with cold PBS. Tissues were then gently shaken in ice-cold 2 mM EDTA PBS at 4 °C for 30 min followed by a vigorous shaking to release villi/crypt epithelia. Subsequently, the supernatant was passed through a sterile 70 μm cell strainer (Corning; 352350). To remove single cells from the filtrate, the pellet was subjected to three rounds of washing in basal culture medium (2 mM GlutaMax, 10 mM Hepes, and 100 U/ml PenStrep in DMEM/F12) and centrifuged at 200*g* for 2 min at 4 °C. The crypt total was calculated and plated at a concentration of 200 crypts/30 μl of Matrigel on an 8-well glass bottom chamber. The crypts were grown in IntestiCult Organoid Growth medium (Stem Cell Technologies; 06005) with the culture medium changed every 24 h. On day 3 of organoid culture, the IntestiCult medium was supplemented with 0.5 μM (Z)-4-hydroxytamoxifen (Sigma; H7904) to induce RAB11A deletion. For embedding the organoids, the Matrigel was dissolved and removed by Corning Recovery Solution (Corning; 354253). Organoids were further centrifuged at 200*g* for 5 min and washed with cold PBS, 2-min centrifugation at 200*g*. Crypts were then resuspended in 4% paraformaldehyde and centrifuged at 200*g* for 5 min followed by a cold PBS wash and resuspension in Matrigel forming clump of organoids and left at 37 °C to solidify. Once solid, 70% ethanol was added and stored at 4 °C until paraffin embedding at the Histology Core of Rutgers Behavioral and Health Science, New Jersey Medical School.

### Immunofluorescence staining

Caco-2 cells (control, RAB11A-KD, and Merlin-KD) grown into a confluent monolayer in four chamber slides (Thermo Fisher Scientific; 154526) and intestinal tissue or organoid samples were fixed with 4% paraformaldehyde or 10% formalin, respectively, and embedded in paraffin. For tissue samples, 5 μm sections were sliced, dewaxed, and subjected to antigen retrieval (0.1 M citric acid; pH 6.0). Both, fixed cell monolayer and intestinal tissue, were then blocked in PBS containing 0.1% Triton-X100, 2% bovine serum albumin, and 2% normal serum for at least 1 h at room temperature followed by incubation with the indicated antibodies overnight at 4 °C. The primary antibodies were rabbit anti-Rab11a (1:200; US Biologicals, R0009), rabbit anti-YAP/transcriptional coactivator with PDZ-binding motif (1:200; CST, 8418), mouse anti-YAP1 (1:400; Novus Biologicals, H00010413-M01), rabbit anti-YAP (1:200; CST, 14074) rabbit anti-α-catenin (1:200; Sigma–Aldrich, C20801, Cell Signaling, 3236), rabbit anti-β-catenin (1:400; Cell Signaling, 9562), mouse anti-β-catenin (1:400; BD Biosciences, 610153), rabbit anti-AMOTL-2 (1:200; Sigma–Aldrich, AV42905), mouse anti-Merlin/NF2 (1:200; Abcam, ab217016), rabbit anti-Merlin/NF2 (D1D8) (1:400; Cell Signaling, 6995), rabbit anti-GRP78 (1:200; Abcam, ab21685), rabbit anti-GM130 (D6B1) (1:200; Cell Signaling, 12480), and mouse anti-E-cadherin (Clone-36) (1:2000; BD Biosciences, 610181). The slides were then washed in cold PBS and incubated with fluorescence-conjugated secondary antibodies for 1 h at room temperature. After incubation, the slides were again washed with PBS followed by 4′,6-diamidino-2-phenylindole counterstaining, before being air dried and mounted with Prolong Gold antifade medium. Images were collected by either LSM 510 laser scanning microscope or Zeiss Observer spinning disk confocal microscope. All images were analyzed by ZEN lite software (version 2.3).

### RAB11A and Merlin coimmunoprecipitation

Human embryonic kidney 293 (HEK293) cells were cultured in DMEM 1×, supplemented with 20% fetal bovine serum, and 1% penicillin–streptomycin. For transfection, the cells were grown to reach 70 to 90% confluence. Followed by DNA plasmid transfection (1 μg) using Lipofectamine 3000 (#L3000008; Invitrogen). The DNA plasmids used were mCherry-tagged RAB11A, enhanced GFP–tagged RAB11A S25V, and HA-tagged Merlin/NF2. Twenty-four hours after transfection, cells were lysed for immunoprecipitation analysis.

### Immunohistochemistry of mouse intestinal tissues

Formalin-fixed paraffin-embedded mouse intestinal tissue sections were dewaxed by heat and xylene treatment followed by hydration of the tissue by a gradient of ethanol ranging from 100 to 70% followed by water. The tissue slides were then quenched for endogenous peroxidase activity by immersing them in methanol with 0.3% hydrogen peroxide solution for 40 min at room temperature. Followed by antigen retrieval using citrate acid–based solution (pH 6.0) for 20 min, during which tissue slides were immersed in the solution and heated using a commercial microwave. The tissue slides were then cooled down 60 °C, washed 1× PBS, and blocked for 1 h at room temperature in PBS containing 2% appropriate animal serum.

Primary antibody incubations were overnight at 4 °C. The primary antibodies used were as follows: mouse anti-β-catenin (BD Laboratory; D10A8) and rabbit anti-YAP (CST; 14074). After primary antibody treatment, the tissue slides were washed in PBS and incubated with biotin-conjugated secondary antibody for 1 h at room temperature. Following the secondary antibody treatment, the slides were incubated with avidin–biotin complex prepared by adding two drops of reagent A and two drops of reagent B (Avidin–Biotin Complex Standard Kit, SK-4100; Vector Labs) in 5 ml of PBS; incubated for 1 h. The tissue slides were then washed in PBS and developed using 3,3′-diaminobenzidine (DAB) solution. The DAB solution was prepared using (DAB Substrate Kit, SK-4100; Vector Labs) 5 ml of double deionized water with two drops of pH 7.4 buffer, four drops of DAB substrate, and two drops of hydrogen peroxide. The tissue slides were then washed and counterstained for nuclei using hematoxylin QS (Vector Labs; H3404). The tissue slides were then washed and dehydrated with ethanol and xylene. The slides were then mounted using CytoSeal 60 (Thermo Fisher Scientific; 8310-4) mounting medium.

### Alcian blue staining

Formalin-fixed paraffin-embedded mouse intestinal sections were deparaffinized by heat and xylene treatment. Following which the sections were hydrated through ethanol gradient to water. Slides were incubated with 3% Alcian Blue 8GX (Sigma; A5268, pH 2.5) for 30 min, washed in distilled water, counterstained by nuclear red, and dried and mounted with Cytoseal 60. Images were collected using a Nikon Eclipse TE2000-U bright field inverted microscope.

### Western blot analysis

Caco-2 (control, RAB11A-KD, and Merlin-KD) and HEK293T cells were grown to monolayers at the time of collection of cell lysates. Cells were washed in cold PBS and lysed with Nonidet P-40 nondenaturing lysis buffer followed by a quick sonication (Microson XL2000) for 10 s twice. The lysis buffer composition and concentration were kept constant as described (39). The lysate was then spun down at maximum speed in a table-top refrigerated microcentrifuge for 10 min, and the resulting supernatant was transferred to a new microcentrifuge tube. Protein concentrations of the lysate were determined using Bradford assay. 20 μg of total protein were loaded onto 8 to 10% SDS-PAGE gels and run at room temperature. Transfer of proteins from gels onto the nitrocellulose membranes was carried out in chilled transfer buffer with methanol set at constant current of 300 mA for 90 min. Membranes were then blocked with 5% milk in Tris-buffered saline with 0.1% Tween-20 for 1 h at room temperature followed by overnight incubation at 4 °C with the desired primary antibodies. The primary antibodies used were as follows: rabbit anti-Rab11a (1:1000; US Biologicals, R0009), rabbit anti-YAP/transcriptional coactivator with PDZ-binding motif (1:1000; CST, 8418), anti-YAP1 (1:2000; Novus Biologicals, H00010413-M01), rabbit anti-YAP (1:1000; CST, 14074), rabbit anti-α-catenin (1:1000; Sigma–Aldrich, C20801; Cell Signaling, 3236), rabbit anti-β-catenin (1:2000; Cell Signaling, 9562), mouse anti-β-catenin (1:2000; BD Biosciences, 610153), mouse anti-E-cadherin (Clone-36) (1:2000; BD Biosciences, 610181), rabbit anti-histone H3 (D1H2) (1:2000; Cell Signaling, 4499), rabbit anti-AMOTL-2 (1:1000; Sigma–Aldrich, AV42905), mouse anti-Merlin/NF2 (1:1000; Abcam, ab217016), rabbit anti-Merlin/NF2 (D1D8) (1:2000; Cell Signaling, 6995), rabbit anti-GRP78 (1:1000; Abcam, ab21685), rabbit anti-GM130 (D6B1) (1:1000; Cell Signaling, 12480), and mouse anti-β-actin (1:2000; Santa Cruz, SC47778). After primary antibody incubation, the membranes were washed for three times in Tris-buffered saline with 0.1% Tween-20. The membranes were incubated with either antimouse or anti-rabbit horseradish peroxidase–conjugated secondary antibody for 1 h. They were further washed and developed using ECL (standard GE Healthcare; RPN2209) or ECL prime (GE Healthcare; RPN2232) detection solution in the dark. Excess ECL solution was drained, and the membranes were placed in a cassette and developed in the dark room using chemiluminescent-sensitive X-ray film.

### Immunoprecipitation assay

Lysates were extracted from Caco-2, RAB11A-KD Caco-2 and Merlin-KD Caco-2 were grown into a monolayer, and HEK293 cells transfected with 3× Flag-RAB11A, 3× RAB11A S25V, and HA-Merlin/NF2 either respectively or simultaneously. Protein G-Agarose (Roche; 11719416001) and anti-FLAG affinity gel (Sigma; A2220) beads used here were prewashed in washed buffer containing 50 mM Tris–HCl (pH 7.5), 150 mM NaCl, 1 mM EDTA, and 1% Triton X-100. With the exception of the FLAG-tagged beads, the beads were incubated overnight with the antibody against the protein of interest. The cell lysates containing 1 mg of total protein were then incubated with the antibody-tagged beads for 6 h at 4 °C and washed three times with wash buffer. The proteins attached to the beads were then eluted by β-mercaptoethanol treatment and heating at 95 °C for 10 min. About 25% of the eluted protein was loaded onto 8% SDS-PAGE gel and run as immunoprecipitation samples.

### Sucrose density gradient centrifugation

Caco-2 and RAB11A-KD Caco-2 cells were grown on 10 cm Petri plates into a monolayer. The cells were lysed in a cold detergent-free lysis buffer followed by a quick sonication (Microson XL2000) for 10 s, twice. The cell lysate extraction buffer composition and their concentration was kept constant as mentioned (80). About 40% and 15% sucrose solutions were prepared and poured simultaneously in ultracentrifuge tube (Beckman coulter; 326819). The tube was then covered with a parafilm and slowly rotated to a horizontal position and left at room temperature for 2 h followed by 2 h in vertical position at 4 °C, forming the sucrose gradient solution. The cell lysate was then gently applied onto the top of the sucrose gradient solution and ultracentrifuged (Beckman Rotor SW55 Ti) at 45,278 rpm at 4 °C for 4 h. Sucrose gradient solution from the top to the bottom of the tube was collected and labeled as fractions 1 to 12. The bottom fraction number 12 corresponds to 40%, and the top fraction number 1 corresponds to a 15%. The aliquots were then prepared and run on SDS-PAGE gels for further assessment of their constituent proteins. The aforementioned procedure was adapted from the respective studies (28, 63).

### TOP-flash reporter assay

The procedure was adapted from the study of Das *et al.* (81). Caco-2 and RAB11A-KD Caco-2 cells were cotransfected with TOP-Flash and *Renilla luciferase* for 24 h. Subsequently, the cells were serum starved for 3 h in DMEM and subsequently treated with Wnt3A (100 ng/ml) (Peprotech; 315-20) for 4 h. Post 4 h of Wnt3A treatment, the cells were lysed, and its luciferase activity was detected using the dual-luciferase assay and Glomax system (Promega). The TOP-Flash activity detected in cell lysates was normalized to the intracellular control, *Renilla luciferase*.

### Statistical analysis

Statistical analyses were conducted using Prism GraphPad 7.04 (GraphPad Software, Inc; https://www.graphpad.com) and Microsoft Excel 2018 software (Microsoft). *p* Values are labeled on individual graphs; *p* < 0.05 was considered as statistically significant. Quantification of Western blots and Pearson's correlation were carried out using ImageJ plug-ins. For Pearson's correlation analysis, perijunctional region was defined as the cell peripheral region marked by junctional proteins, and nuclear region was defined by 4′,6-diamidino-2-phenylindole staining. The “coloc2” function of the ImageJ software Pearson's correlation was used to calculate the correlation between two proteins within a manually demarcated region of interest. Data are represented as bar graphs with mean ± SEM.

## Data availability

All data have been included within the article.

## Supporting information

This article contains supporting information.

## Conflict of interest

J. R. G. reports of receiving commercial research grant from ViiV, Inc. All other authors declare that they have no conflicts of interest with the contents of this article.
