# Supplementary material for: RAB11A-mediated YAP localization to adherens and tight junctions is essential for colonic epithelial integrity
Source: J Biol Chem. 2021 May 29;297(1):100848. doi: 10.1016/j.jbc.2021.100848 (PMC8254046; doi:10.1016/j.jbc.2021.100848)
Supplement: Supplemental Figures S1–S5 [file mmc1.pdf]

RAB11A-mediated YAP localization to adherens and tight junctions is essential for colonic epithelial integrity

Goswami et al.

Supporting Data:

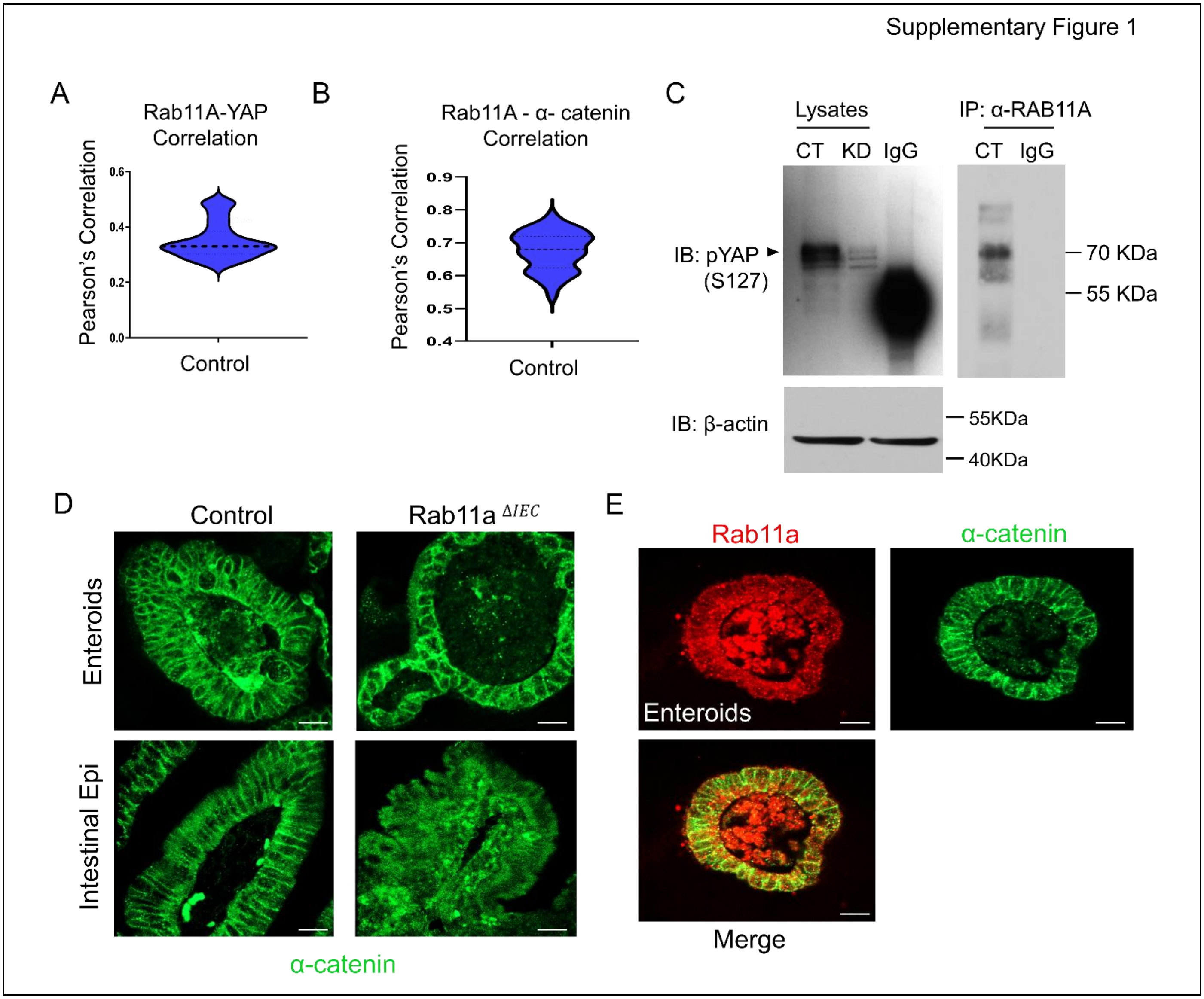

Supplementary Figure 1.
A. Pearson's correlation between RAB11A and YAP in control Caco2 cells. r = 0.356. **B.** Pearson's correlation between RAB11A and α-catenin. r = 0.67. **C.** Co-IP assay between pYAP S127 and RAB11A. RAB11A-KD cells exhibited a diminished pYAP S127. Co-IP showed biochemical association between RAB11A and pYAP S127. IgG serves as a control. **D.** Immunofluorescent staining of α-catenin (in green) in WT and Rab11a-deficient mouse enteroids and IECs. **E.** Immunofluorescent staining of Rab11a and α-catenin in wild type enteroids. Data are representative of three independent experiments**.** Scale bars, 10 µm.

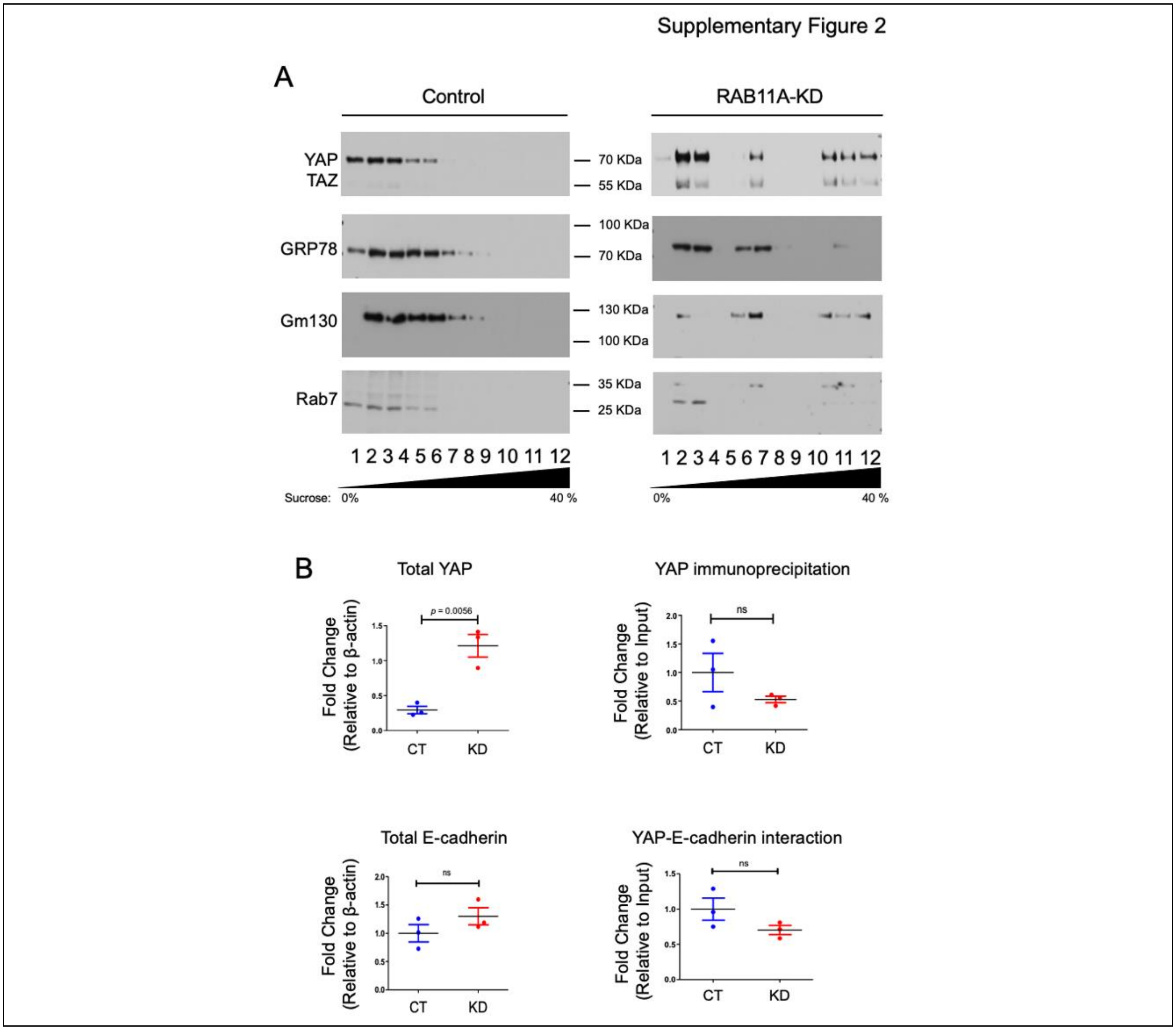

Supplementary Figure 2.
A. Sucrose gradient cell fractionation shown here with a gradient ranging from 0-40% corresponding to lanes 1-12 in both control and RAB11A-KD cells. Fractions were resolved and blotted for Gm130 (a Golgi body marker), GRP78 (an endoplasmic reticulum marker) and Rab7 (a lysosome marker). Note that RAB11A depletion had an impact on general membrane trafficking machinery. YAP/TAZ western blot is from Fig 2A. **B.** Quantifications for total YAP and E-cadherin as well as their interaction in control and RAB11A-KD cells were from three independent experiments.

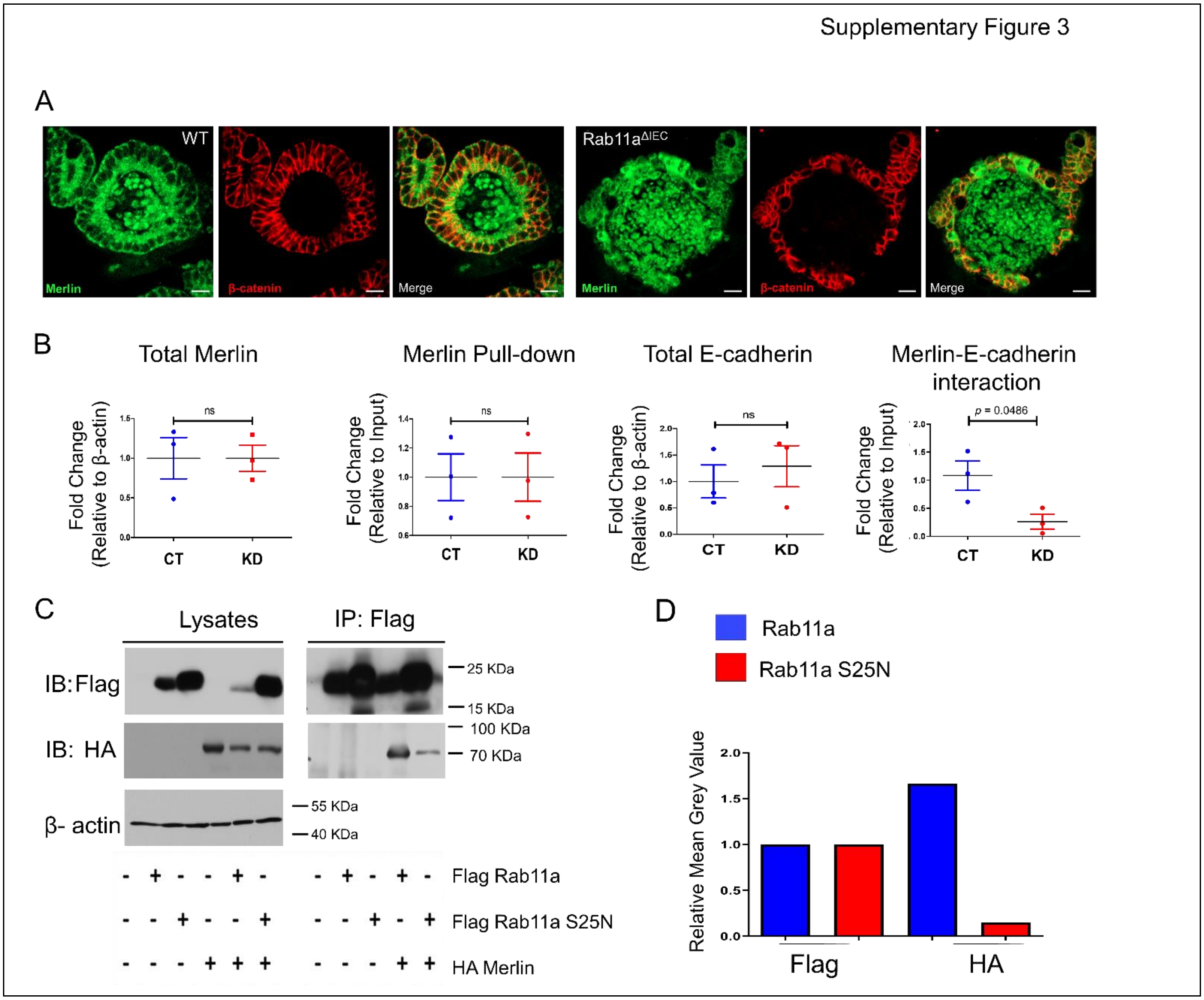

Supplementary Figure 3

A. Confocal immunofluorescent staining for Merlin (green) and β-catenin (red) in wild type and Rab11a-deficient mouse enteroids. **B.** Quantification of total Merlin, E-cadherin, and their interactions in control and RAB11A-KD cells was from three independent experiments. **C-D.** Co-IP assay between FLAG-Rab11a or FLAG-Rab11a (S25N) with HA-Merlin was performed in HEK293T cells and quantified. Scale bars, 10 μm.

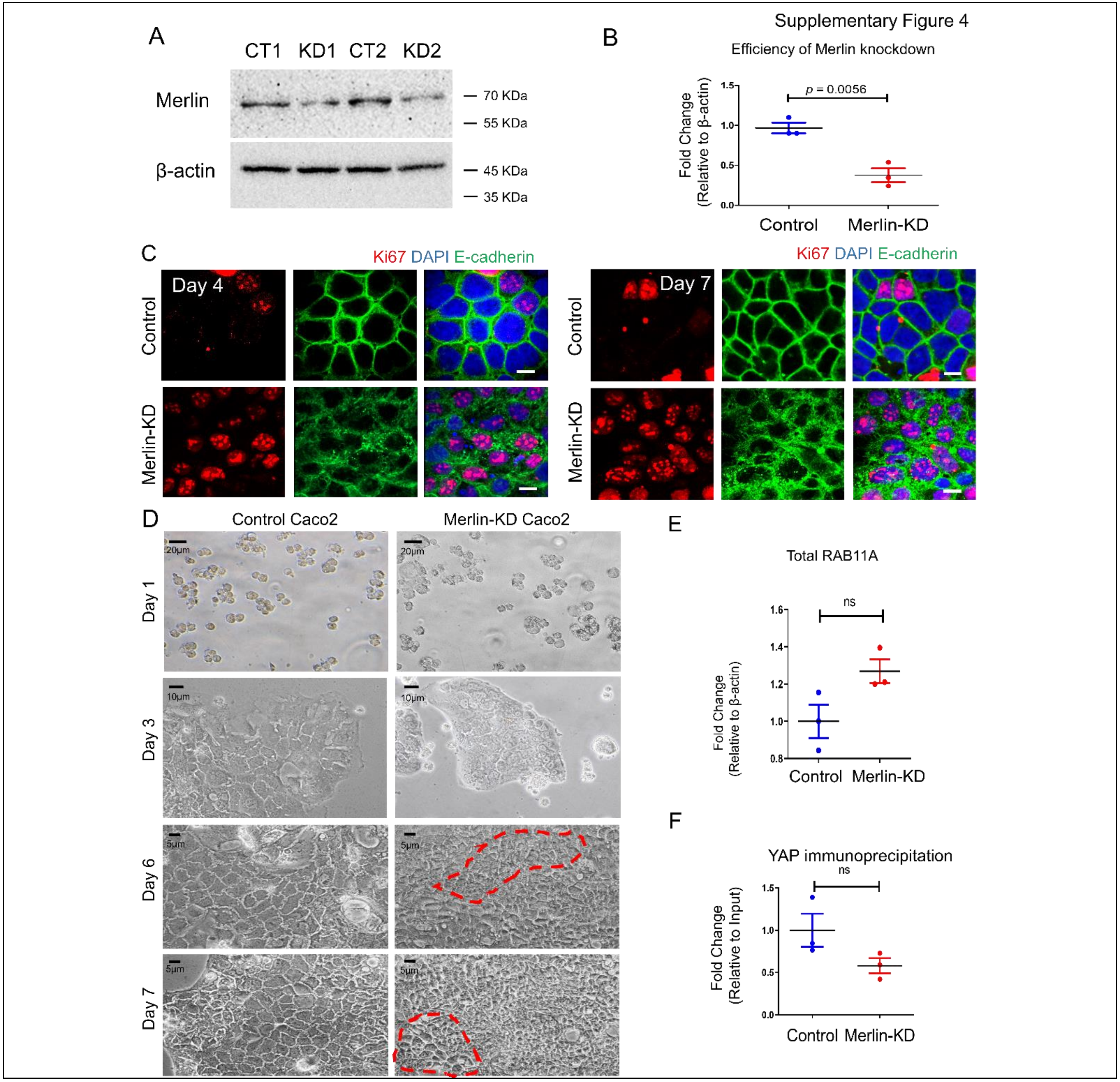

Supplementary Figure 4

A. Efficiency of Merlin knockdown in independently selected stable Caco2 lines. **B.** Quantification of the efficiency of Merlin knockdown. **C.** Immunostaining for Ki67 in control and Merlin-KD cells on day 4 and 7. Separate channels of image provided in Fig 4B. Scale bars, 10 µm. **D.** Representative bright field images of control and Merlin-KD Caco2 cells on day 1, 3, 6, 7. Cells were plated as singlet. On days 6 and 7 Merlin-KD cells appeared to grow on top of each other (red-outlined regions). **E-F.** Quantification of total RAB11A and immunoprecipitated YAP in control and Merlin-KD cells (3 independent experiments).

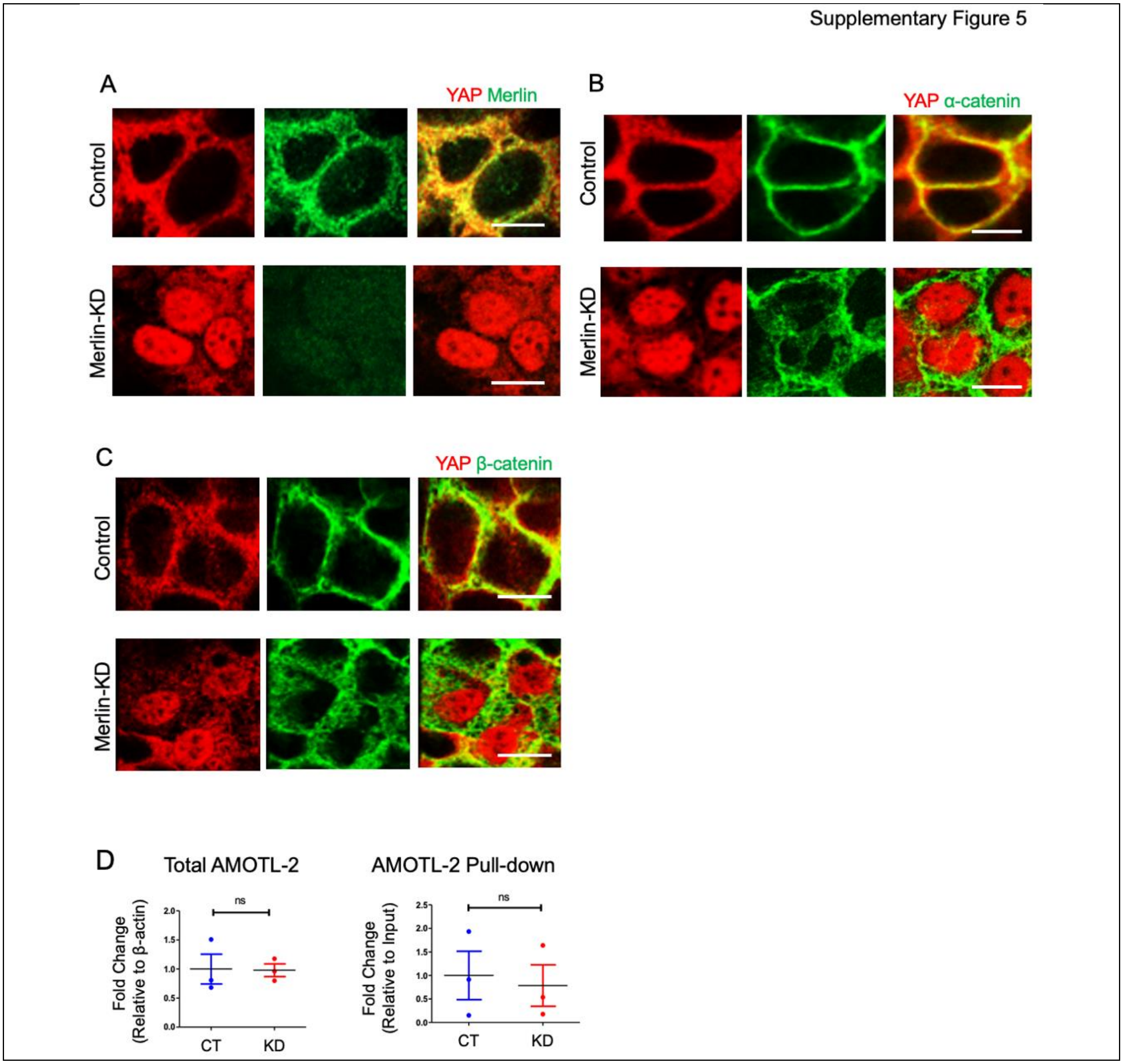

Supplementary Figure 5

A. Immunofluorescent staining of YAP (red) and Merlin (green) in control and Merlin-KD cells. **B.** Immunofluorescent staining of YAP (red) and α-catenin (green) in control and Merlin-KD cells. **C.** Immunofluorescent staining of YAP (red) and β-catenin (green) in control and Merlin-KD cells. **A-C.** Separate channels of image provided in Fig 4G **D.** Quantification of total AMOTL-2 and immunoprecipitated AMOTL-2 in control and RAB11A-KD cells. Scale bars, 10 µm.
